# Effects of Clavulanic Acid on the Acquisition and Reinstatement Following Morphine-induced Conditioned Place Preference in Mice

**DOI:** 10.32598/bcn.9.4.289

**Published:** 2018-07-01

**Authors:** Soghra Mehri, Seyed Saber Sajjadi, Seyed Meghdad Tabatabai, Hossein Hosseinzadeh

**Affiliations:** 1. Pharmaceutical Research Center, Pharmaceutical Technology Institute, Mashhad University of Medical Sciences, Mashhad, Iran.; 2. Department of Pharmacodynamics and Toxicology, School of Pharmacy, Mashhad University of Medical Sciences, Mashhad, Iran.

**Keywords:** Clavulanic acid, Morphine, Glutamic acid, Memantine

## Abstract

**Introduction:**

β-Lactam antibiotics like Clavulanic Acid (CA) enhances cellular glutamate uptake through activation of Glutamate Transporter subtype 1 (GLT-1) and decreases the level of glutamate in the nervous system. Based on studies, blocking the glutamate activity inhibits morphine-induced Conditioned Place Preference (CPP) in animals. Therefore, the effects of CA on the acquisition of morphine craving were evaluated using the CPP model in the current study.

**Methods:**

CA (1, 50 and 150 mg/kg, ip) was co-administered with morphine (40 mg/kg) for 4 days in the conditioning phase. On day 8, the effects of CA on morphine preference was assessed. In another experiment, the effect of CA on reinstatement of morphine preference by a single morphine injection (10 mg/kg) was evaluated after an extinction period.

**Results:**

In the first method, the morphine-induced place preference was markedly reduced following administration of CA (50 and 150 mg/kg). In the second experiment, a single administration of CA (50 and 150 mg/kg) markedly inhibited the reinstatement of morphine preference on day 16. The results indicated that CA (50, 150 mg/kg) can block both morphine-induced CPP and the reinstatement of place preference following priming dose of morphine. Also memantine (as a positive control) (10 mg/kg) significantly inhibited both acquisition and reinstatement of morphine CPP.

**Conclusion:**

Considering the important role of glutamate neurotransmission in morphine dependence, the effects of CA may be partly due to decrease in glutamate level in synaptic space and blockade of N-Methyl-D-aspartate Acid (NMDA) receptors. Although, we need further studies to determine exact cellular mechanism.

## Highlights

- Clavulanic acid inhibits morphine-induced place preference in mice.
- Clavulanic acid blocks morphine-induced reinstatement in mice.
- Single and multiple administration of clavulanic acid inhibits morphine-induced CPP.

## Plain Language Summary

Opiate dependence is a chronic disorder and needs long-term treatment to prevent relapse in abstinent individuals. Research on safe drugs, which can block opiate dependence seems to be necessary. Clavulanic acid is a β-lactam antibiotic with good oral bioavailability, low antibiotic activity and good CNS (Central Nervous System) penetration. Regarding the effect of CA in CNS, in the current study, the effects of different doses of this compound on the acquisition of morphine craving were evaluated using the CPP (conditioned place preference) model in mice. For this purpose, two model were used. In the first one, the effect of CA of morphine preference was mentioned. In the second model, we evaluated how reinstatement of morphine preference can be affected following single administration of CA. The obtained results exhibited that multiple administration of CA (50 and 150 mg/kg, ip) markedly inhibited morphine preference in mice. Additionally, single administration of CA (50 and 150 mg/kg, ip) can block reinstatement of morphine. Given the important role of glutamate neurotransmitter in morphine dependence, the effect of CA in this study, at least in part, is due to modulation of this neurotransmitter.

## 1. Introduction

Opiate dependence is a chronic disorder which needs long-term treatment with focus on the prevention of relapse in abstinent individuals (McLellan, Lewis, O’Brien & Kleber, 2000). Drug seeking and craving in abstinent individuals can be observed following exposure to environmental cues such as places of drug consumption (Shalev, Grimm & Shaham, 2002). Several neuroanatomical systems are involved in morphine dependence. The mesolimbic pathway (the reward pathway) is a dopaminergic pathway which has important role in the rewarding actions of opiates (Wise, 2002). Other neurotransmission systems including GABA (gamma-aminobutyric acid) and glutamate have exhibited modulatory effects in development of dependence phenomena (Kalivas & Volkow 2011; Tan, Rudolph, & Lüscher, 2011; Zarrindast et al., 2004).

It has been shown that glutamate-related plastic changes in the circuitry from the Prefrontal Cortex (PFC) to the nucleus accumbens are important for drug relapse (Kalivas & Volkow 2011). Additionally, the astrocytes mainly modulate glutamate dynamics via controlling the activities of the catalytic subunit of the Cysteine-glutamate exchanger (xCT) and Glutamate transporter 1 (GLT-1) (Reissner & Kalivas, 2010). In agreement with these findings, memantine as a NMDA (N-methyl-D-aspartate) receptor antagonist significantly prevented morphine–induced Conditioned Place Preference (CPP) in mice (Do Couto et al., 2004; Popik, Wrobel & Bisaga, 2005).

Moreover, oxidative/nitrosative pathways demonstrated an important role in opiate addiction (Abdel-Zaher, Abdel-Rahman & Elwasei, 2010). The induction of apoptosis in hypothalamus and hippocampus during CPP is another underlying mechanism which is involved in morphine CPP (Haghparast et al., 2013). Therefore, the treatment of opiate dependence can be achieved at least in part through disruption of discussed mechanisms.

The CPP paradigm is a valid animal model extensively used to evaluate the mechanisms underlying context-dependent learning associated with drug abuse (Bardo, Rowlett & Harris, 1995). Clavulanic Acid (CA) is previously known as a non-competitive inhibitor of β-lactamase. This compound itself has negligible antibiotic activity and is normally given in combination with some beta-lactam antibiotics like ticarcillin and amoxicillin to overcome resistance in bacteria which can secrete the β-lactamase enzyme (Chen et al., 2013). Oral bioavailability, low antibiotic activity and good CNS (central nervous system) penetration are important properties of CA already mentioned in neuropharmacology (Bolton et al., 1986; Nakagawa et al., 1994).

Several lines of evidence demonstrated neuroprotective effects of CA. This compound exhibited strong anxiolytic activity in rodents and cotton-top tamarins with minimal side effects (Kim et al., 2009). Anti-inflammatory (Banani et al., 2012), anticonvulsant (Chen et al., 2013), antinociceptive (Hajhashemi & Dehdashti, 2014) and stimulatory effect on sexual behaviors (Chan et al., 2009) are reported following administration of CA in different laboratory animals models. CA strongly protected neurons which exposed to MPTP (1-methyl-4-phenyl-1,2,3,6-tetrahydropyridine) or kainic acid as potent neurotoxic agents (Huh et al., 2010). The exposure of dopaminergic cells to MPP (1-methyl-4-phenylpyridinium) markedly elevated ROS (Reactive Oxygen Spices) and induced apoptosis in treated cells (Kost et al., 2012). Additionally, CA attenuated the rewarding, hyperthermic, and locomotor-sensitizing effects of morphine in rats (Schroeder et al., 2014). Interestingly, administration of CA could not prevent development of tolerance and dependence to morphine in mice (Hajhashemi & Dehdashti, 2014).

As mentioned above, among different neurotransmitters involved in morphine-induced CPP, the glutamate pathway has an important role. GLT1 can modulate glutamate uptake in the brain and termination of glutamatergic transmission (Danbolt, 2001). Studies reported β-lactam antibiotics can increase GLT1 activity and consequently diminish level of synaptic glutamate (Rothstein et al., 2005; Chen & Wang, 2012). Ceftriaxone as a β-lactam antibiotic elevated the synthesis and membrane insertion of GLT1. Interestingly, restoring GLT1 with ceftriaxone prevented reinstated cocaine seeking in animals (Knackstedt et al., 2010). Based on neuromodulatory activity of CA, the inhibition of ROS production and apoptosis which are involved in morphine CPP, we decided to evaluate the effects of CA on acquisition and reinstatement of morphine-induced CPP in mice.

## 2. Methods

### 2.1. Materials

Clavulanate potassium was a gift from Daana Pharmaceutical Co. (Tabriz, Iran). Morphine sulfate was purchased from Daru Pakhsh. (Iran). Drugs were dissolved in Normal Saline (NS) 0.9% and administrated intraperitoneally (ip).

### 2.2. Animals

Male Razi mice, 25–30 g were housed in colony rooms with 12.12 h light/dark cycle at 21±2°C with free access to food and water.

### 2.3. CPP apparatus

The CPP apparatus had three compartments. Two main compartments of the apparatus (compartments A and B) with equal size (30-length×30-width×35-height) and different colored walls (black vs. white). These two compartments had different floor textures; fine grid in black section and wide grid in white section. The compartments A and B were separated by a small and gray central compartment during the test (Hosseini, Nemati Karimooy & Alaei, 2008; Imenshahidi, Zafari & Hosseinzadeh, 2011).

### 2.4. Experimental procedure

#### 2.4.1. Acquisition of place preference

**Figure 1.**
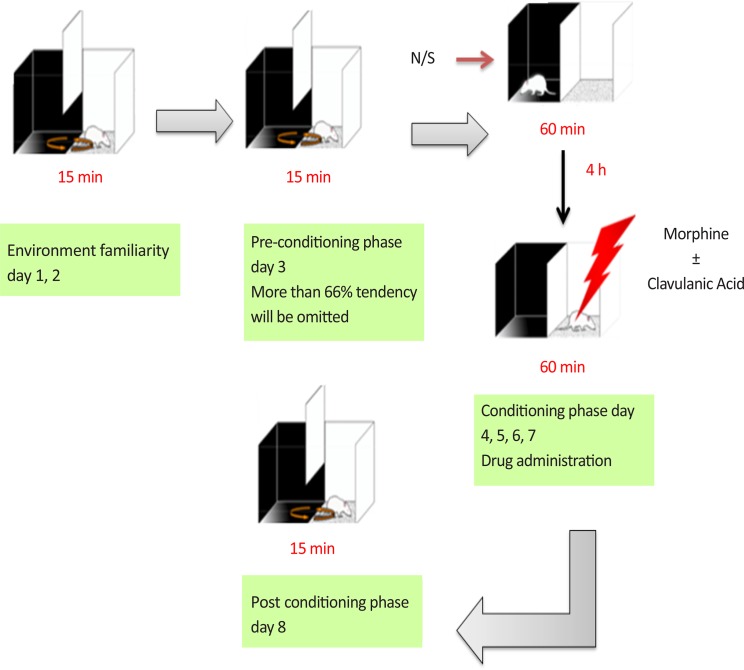
Schematic representation of acquisition of morphine-induced place preference

As shown in Figure 1, the experiment consisted of an eight-day schedule with three phases. The animals were divided into 7 groups (n=6): Norma saline, Morphine (40 mg/kg), Morphine (40 mg/kg)+CA (1, 50 and 150 mg/kg), CA (50 mg/kg), Morphine (40 mg/kg)+memantine (10 mg/kg).

In the first phase (pre-conditioning), the mice move freely in both compartments of the apparatus for 15 min each day for 2 days. On day 3, all the time spent by the animal in each compartments A and B for 15 min were measured. The animals with strong unconditioned preference (more than 66% of the session time) for any compartment were excluded. During the second phase (conditioning), on days 4–7, the animals received normal saline (ip) and were placed in black compartment for 1 h. After 4 h, they received the drugs (ip) and were confined in the white compartment.

In the third phase (post-conditioning) on day 8, the partition was removed and the time which spent by the animals in drug paired compartments (A and B) was recorded for 15 min. The time spent in the central gray was proportionally divided between both conditioning compartments (Do Couto et al., 2004; Imenshahidi, Zafari & Hosseinzadeh, 2011).

### 2.4.2. Extinction and reinstatement

**Figure 2.**
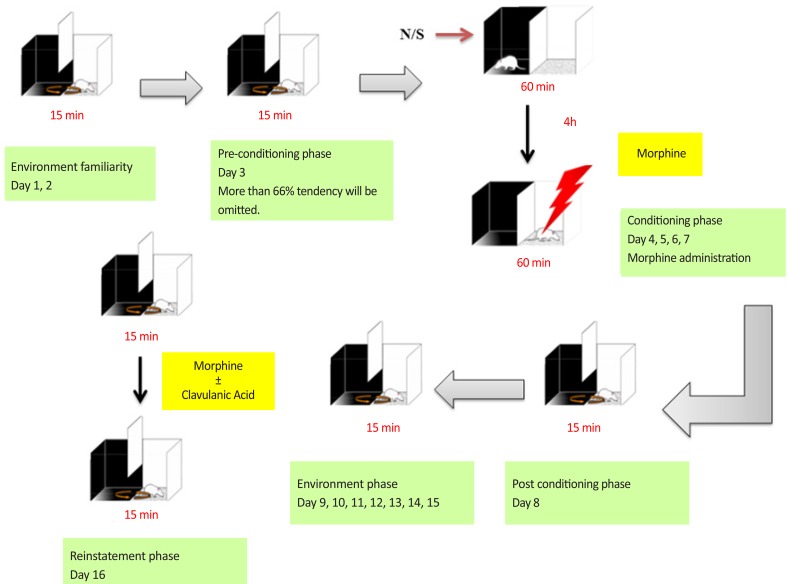
Schematic representation of extinction and reinstatement of place preference

At the first step, 3 phases that described above were considered. Then for evaluation the effect of CA on the reinstatement of morphine-induced CPP the procedure was continued. On days 8–15 for extinction phase, animals were placed in the apparatus with free access for 15 min to both compartments without any drug administration. On day 15, time spent in the white compartment for each group of animals was similar to those of pre conditioning phase. On the day 16, after the last extinction session, CA (1, 50 and 150 mg/kg) was administrated. After 30 min, for induction of reinstatement, animals were given single dose of morphine (10 mg/kg). Finally the preference was measured by giving the animals, free access to both sides of the experimental box for 15 min (Do Couto et al., 2003; Do Couto et al., 2005; Imenshahidi, Zafari & Hosseinzadeh. 2011). It should be noted that the animals that received CA in reinstatement day didn’t receive CA during conditioning phase. The schematic of extinction and reinstatement of place preference has been shown in Figure 2.

### 2.5. Statistical analysis

Data were presented as mean±SEM. Statistical analyses were performed with two-way analysis of variance (ANOVA), followed by Bonferroni test. P-value less than 0.05 (P<0.05) was considered to be statistically significant.

## 3. Results

### 3.1. Effects of CA on the acquisition of morphine-induced CPP

Two-way ANOVA, followed by Bonferroni’s test [treatment effect: F_(1, 84)_=8.52, P<0.0001, dose effect F_(6, 84)_=8.48, P<0.0001, treatment×dose interaction: F_(6, 84)_=10.03, P<0.0001 indicated that pretreatment with CA attenuated the acquisition of morphine-induced CPP.

**Figure 3.**
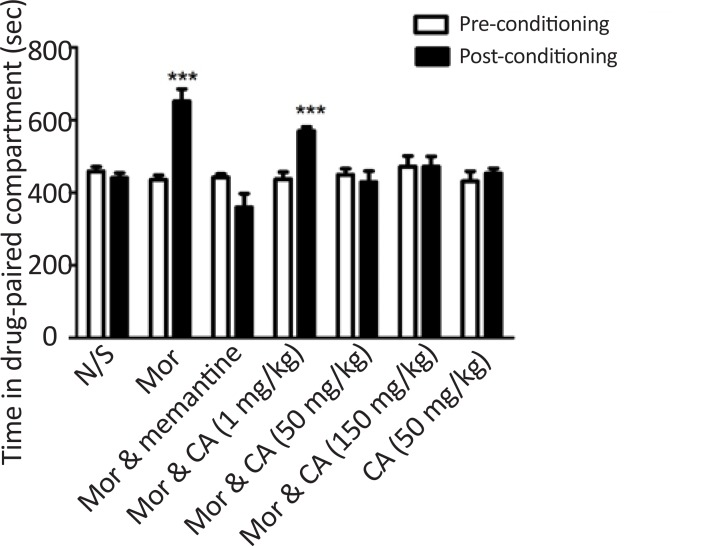
Effects of CA (1, 50 and 150 mg/kg) on the acquisition of morphine-induced CPP in mice During the conditioning phase, animals were treated with different doses of CA in the drug-paired compartment. Data are shown as mean±SEM of 6 animals per group. The bars display the time spent in the drug-paired compartment before conditioning sessions in pre-conditioning test and after conditioning sessions in post-conditioning test. ***P<0.001 significant differences between the time spent in the drug-paired compartment in pre-conditioning vs. post-conditioning sessions tests; CA: Clavulanic Acid; Mor: Morphine.

As shown in Figure 3, administration of morphine significantly increased the time spent in the white compartment in post conditioning phase (P<0.001) which means morphine could induce place preference. CA (50 and 150 mg/kg) markedly reduced the difference in occupancy time in morphine-paired compartment during pre-conditioning and post-conditioning phase. Following administration of CA alone, place preference was not observed. Memantine (10 mg/kg) as a positive control significantly inhibited morphine-induced CPP.

### 3.2. Effects of CA on reinstatement of morphine-induced CPP

During days 8–16, daily extinction session could disappear animals conditioning which induced by morphine (40 mg/kg). No significant difference between pre-conditioning and the extinction session of each group was observed. In reinstatement tests, following administration of 10 mg/kg morphine, the animals spent significantly more time in the drug-paired compartment when compared to pre-conditioning phase (P<0.001).

**Figure 4.**
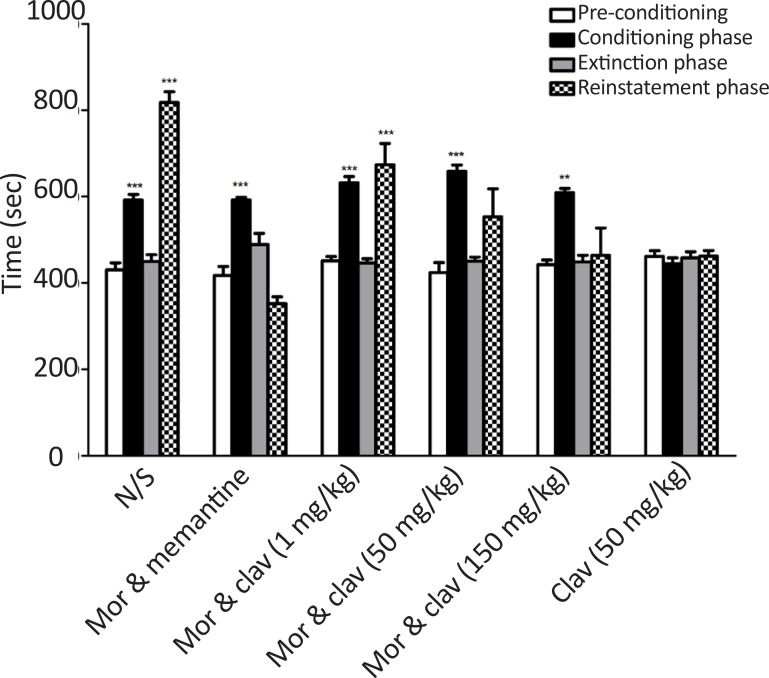
Effects of CA (1, 50 and 150 mg/kg) on the reinstatement of morphine CPP caused by morphine (10 mg/kg) Data are shown as mean±SEM of 6 animals per group. The bars display the time spent in the drug-paired compartment before conditioning sessions (white bars), after conditioning sessions (black bars), in the last extinction session (dark grey bars) and in the reinstatement test. ***P<0.001; **P<0.01 significant difference between the time spent in pre-conditioning vs. post-conditioning sessions or reinstatement tests; CA: Clavulanic Acid; Mor: Morphine.

Two-way ANOVA, followed by Bonferroni’s test [treatment effect: F_(3, 144)_=48.60, P<0.0001, dose effect F_(5, 144)_=13.84, P<0.0001, treatment×dose interaction: F_(15, 144)_=13.03, P<0.0001 indicated that pretreatment with CA attenuated the morphine reinstatement. As shown in Figure 4, administration of CA (1 mg/kg) couldn’t block morphine reinstatement (P<0.001 vs pre-conditioning) while CA (50 and 150 mg/kg) significantly inhibited reinstatement of morphine. Also, memantine (10 mg/kg) inhibited morphine reinstatement in the current study.

## 4. Discussion

Our results were consistent with other study results in which the animals exhibited an obvious preference for the environment, which paired with morphine administration (Tzschentke, 1998; Do Couto et al., 2003). Our results also demonstrated that systemic administration of CA reduced the acquisition and reinstatement of morphine-induced CPP. To evaluate possibility that CA had rewarding effect, a separate group of animals was evaluated in CPP paradigm without morphine administration. Results showed CA had no rewarding effect.

Different neurotransmitter pathways are involved in morphine induced CPP. Among them, glutamate neurotransmission has shown a critical role in several studies (Do Couto et al., 2004; Do Couto et al., 2005). Memantine, a NMDA antagonist, markedly inhibited the acquisition and reinstatement of morphine CPP in animals (Do Couto et al., 2004; Popik, Wrobel & Bisaga, 2005; Do Couto et al., 2005). The electrical stimulation of the hippocampal glutamate/NMDA fibers or micro-injection of NMDA into the VTA induces reinstatement of cocaine-seeking behavior (Vorel et al., 2001). The modulation of glutamate hemostasis is performed by glutamate uptake through an efficient and high capacity glutamate transporter system. GLT-1 has been considered as an important transporter in the regulating of glutamate uptake (Danbolt, 2001). Based on reports, β-lactam antibiotics can increase GLT1 activity and consequently diminish the level of synaptic glutamate. Therefore, in the current study, CA effects in inhibition of acquisition and reinstatement of morphine–induced CPP may be partially achieved through modulation of glutamate neurotransmitter.

Dopaminergic system is another important pathway which is involved in rewarding effects of morphine. Dopamine antagonists (haloperidol, clozapine, risperidone and SCH 23390) could reverse morphine CPP in mice (Manzanedo et al., 2001). Also it was reported that dopamine antagonist failed to block reinstatement of morphine CPP (Do Couto et al., 2005). Other studies showed dopamine transmission can be increased following CA administration (Chan et al., 2009). In our study, CA inhibited both acquisition and reinstatement of morphine-induced CPP. Therefore, the lack of protective effect of dopamine antagonist on reinstatement of morphine CPP and effect of CA on augmentation of dopamine transmission suggested that inhibitory effect of CA on acquisition and reinstatement of morphine CPP may be regulated mainly throughout glutamate pathway and not dopamine transmission. Although, because we didn’t evaluate the cellular and molecular mechanism of CA on morphine CPP, further studies need to determine the exact effect of this compound on both dopamine and glutamate pathways.

The induction of apoptosis and oxidative stress pathways are another suggested mechanisms involved in morphine CPP (Abdel-Zaher, Abdel-Rahman & Elwasei, 2010; Haghparast et al., 2013). The level of proteins which are involved in apoptosis pathway (Bax, Bcl-2, PARP and caspase 3) significantly changed in morphine induced CPP models in rat (Haghparast et al., 2013). In regard to neuroprotective effect of CA, treatment of SH-SY5Y cells with this compound markedly decreased MPP caused cytotoxicity especially via reduction of ROS production and inhibition of apoptosis (Kost et al., 2012). Therefore, the reduction oxidative/nitrosative pathway and regulation of apoptosis death following CA administration can be considered another alternative mechanism.

Locomotor activity test was not done in this study. Other behaviors, including locomotor activity, could also influence the results in the CPP paradigm, yielding false-positive or negative results (Huston et al., 2013), therefore it was better to evaluate the locomotor activity. However, it has been reported CA (1 mg/kg) did not change locomotor activity in rats (Schroeder et al., 2014). The results showed administration of CA can block both acquisition and reinstatement of morphine induced CPP in mice. It seems that this effect of CA in part may be due to the effect on GLT1 activity and glutamate pathway.

## Ethical Considerations

### Compliance with ethical guidelines

All study experiments were done according to Ethics Committee Acts of Mashhad University of Medical Sciences (Ethics code: 910623).
